# Diagnostic ability of multifocal electroretinogram in early multiple sclerosis using a new signal analysis method

**DOI:** 10.1371/journal.pone.0224500

**Published:** 2019-11-08

**Authors:** L. Boquete, E. López-Guillén, E. Vilades, J. M. Miguel-Jiménez, L. E. Pablo, L. De Santiago, M. Ortiz del Castillo, M. C. Alonso-Rodríguez, E. M. Sánchez Morla, A. López-Dorado, E. Garcia-Martin

**Affiliations:** 1 Biomedical Engineering Group, Electronics Department, Universidad de Alcalá, Alcalá de Henares, Madrid, Spain; 2 RETICS: Thematic Networks for Co-operative Research in Health for Ocular Diseases, Madrid, Spain; 3 Ophthalmology Department, Miguel Servet University Hospital, Zaragoza, Spain; 4 Aragon Institute for Health Research (IIS Aragon), Innovative and Research Group Miguel Servet Ophthalmology (GIMSO), University of Zaragoza, Zaragoza, Spain; 5 Physics and Mathematics Department, Universidad de Alcalá, Alcalá de Henares, Madrid, Spain; 6 Institute for Health Research 12 de Octubre Hospital (i+12), Madrid, Spain; University of Rochester Medical Center, UNITED STATES

## Abstract

**Purpose:**

To determine if a novel analysis method will increase the diagnostic value of the multifocal electroretinogram (mfERG) in diagnosing early-stage multiple sclerosis (MS).

**Methods:**

We studied the mfERG signals of OD (Oculus Dexter) eyes of fifteen patients diagnosed with early-stage MS (in all cases < 12 months) and without a history of optic neuritis (ON) (F:M = 11:4), and those of six controls (F:M = 3:3). We obtained values of amplitude and latency of N1 and P1 waves, and a method to assess normalized root-mean-square error (F_NRMSE_) between model signals and mfERG recordings was used. Responses of each eye were analysed at a global level, and by rings, quadrants and hemispheres. AUC (area under the ROC curve) is used as discriminant factor.

**Results:**

The standard method of analysis obtains further discrimination between controls and MS in ring R3 (AUC = 0.82), analysing N1 waves amplitudes. In all of the retina analysis regions, F_NRMSE_ value shows a greater discriminating power than the standard method. The highest AUC value (AUC = 0.91) was in the superior temporal quadrant.

**Conclusion:**

By analysing mfERG recordings and contrasting them with those of healthy controls it is possible to detect early-stage MS in patients without a previous history of ON.

## Introduction

The multifocal electroretinogram (mfERG) technique obtains objective and qualitative measurements regarding the functioning of the retina excited with different types of visual stimuli, and was developed for the simultaneous detection of the electrical activity from specific sectors of the retina [1]. A major advantage of the mfERG technique is that it reveals bipolar cell functionality (mainly) and photoreceptor contribution, as well as the spatial distribution of the aforementioned sensitivity [2].

In the mfERG recordings, the retina is divided into a defined number of sectors with hexagonal shape (61 or 103). In the ISCEV standard stimulus array is scaled to elicit comparable response amplitudes from each stimulus sector, resulting in larger hexagons with increasing eccentricity [3]. Visual stimulation between black and white in each hexagon is regulated by a pseudo-randomized sequence (m-sequence) [4]. It is possible to obtain an individualized response in each sector through the correlation between the continuous signal registered in the electrodes and the m-sequence.

The standard stimulus of the mfEGR provides the first-order kernel (FOK), a response to a dark-to-light or light-to-dark stimulus. The response in the FOK comes from the receptor cells and the “on” or “off” bipolar cells in each hexagon [1].

**Fig 1:**
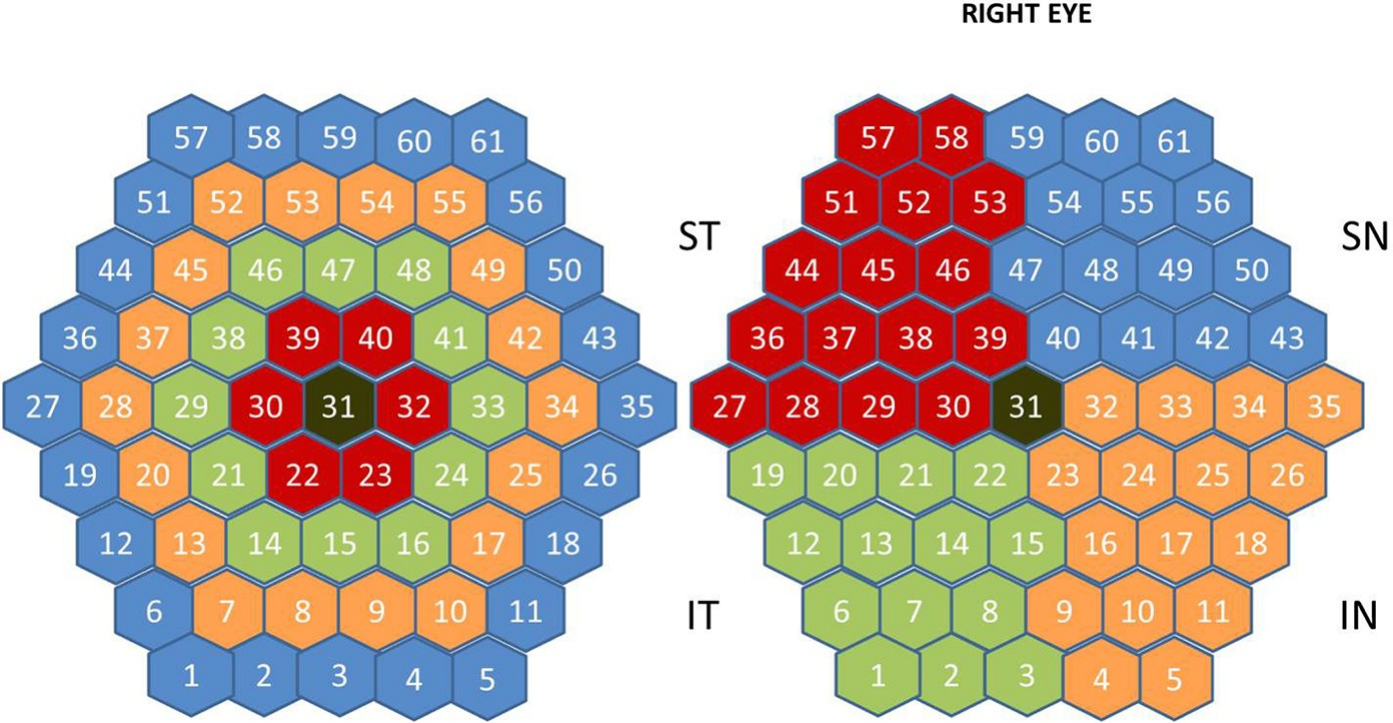
Stimulated visual field. In this figure the hexagons are not scaled with retinal eccentricity. (a) Definition of the rings (R1, … R5). (b) Definition for OD of the quadrants: ST, IT, SN, IN.

In past studies, the diagnostic measurements most commonly taken in mfERG recordings were the amplitudes and latencies of N1 and P1 waves [5]. It is possible to form groups (Fig 1) of answers from the trace arrays of the full visual field (SUM), rings (R1…R5), quadrants (ST: superior temporal, IT: inferior temporal, IN: inferior nasal, SN: superior nasal) and hemiretinal areas: SH (superior hemifield = ST+SN), IH (inferior hemifield = IT+IN), TH (temporal hemifield = ST+IT) and NH (nasal hemifield = IN+SN).

Multiple sclerosis (MS) is a neurodegenerative disease characterized by chronic demyelination of the central nervous system and which, as it develops, severely compromises patients’ quality of life. There is no single biomarker which is valid for diagnosis of MS; several are used instead, including clinical diagnosis, magnetic resonance imaging (MRI), cerebrospinal fluid data and evoked potentials. MRI can be considered the most important tool to diagnose and monitor multiple sclerosis [6]. However, the last review of the McDonald criteria [7] highlighted the need to perform further research into optic nerve involvement, validation in diverse populations and incorporation of advanced imaging and neurophysiological and body fluid markers. Evoked potentials are more closely related to clinical disability than structural data [8], making it possible to anticipate clinical deterioration based on the high correlation observed with the Expanded Disability Status Scale [9], even in progressive forms of MS [10].

The aim of this study is to analyse the ability of mfERGs to diagnose MS at an early stage of the disease. Two methods of analysis were investigated; the first one based on standard measures of amplitudes and latencies of N1 and P1 waves; the second method consists of building a signal model from subject control recordings and comparing these under assessment with this model.

## Methods

The study procedures were performed in accordance with the tenets of the Declaration of Helsinki, and the study protocol was approved by the local ethics committees [Clinical Research Ethic Committee of Aragon (CEICA, Zaragoza, Spain)]. Written informed consent to participate in the study was obtained from all subjects.

Recordings were acquired in the Ophthalmology Service of the University Hospital Miguel Servet (Zaragoza, Spain). mfERG signals of both eyes in 15 subjects (F:M = 11:4) with early diagnosis (inferior to 12 months) without previous history in optic neuritis and 6 healthy controls (F:M = 3:3) were used. The mean age (SD) was 44.47 (8.24) years for the patients and 35.75 (10.57) years for the controls. There was no significant difference between the two groups with respect to age.

MS was diagnosed based on the 2010 revision of the McDonald Criteria [11] confirmed by a neurologist specializing in MS. Patients with a visual acuity of less than 20/32 on the Snellen scale or 0.2 on the ETDRS scale, intraocular pressure > 20 mmHg, and/or active MS flare (of any neurologic deficit) in the 3 months preceding their enrolment into the study or at any of the annual visits were excluded from the study. Active MS flare was considered a reason for exclusion because acute axonal loss could mask neuronal damage secondary to MS progression (i.e., chronic neurodegeneration), which was the main purpose of this study.

The participants had no concomitant ocular diseases, nor any previous history of retinal pathology, glaucoma, amblyopia or significant refractive errors (more than 5 dioptres of spherical equivalent refraction or 3 dioptres of astigmatism), or systemic conditions that could affect the visual system.

A complete neuro-ophthalmic examination, including assessment of best-corrected visual acuity using the Snellen and ETDRS charts, contrast sensitivity with CSV1000 test, colour vision with Ishihara, pupillary reflexes, ocular motility; examinations of the anterior segment, intraocular pressure (IOP) with the Goldmann applanation tonometer, and papillary morphology by funduscopic exam was performed in all subjects in order to detect any ocular alteration (such as primary open angle glaucoma, cataract, corneal pathology) that may affect functional vision or mfERG results.

The first order mfERG kernel was obtained according to the ISCEV standard [5] using a Roland Retiscan system. The stimulus configuration used was the 61 hexagon array (Fig 1) scaled with eccentricity (the area of the hexagons increases towards periphery to compensate for lower cone density).

A monocular recording of both eyes was carried out randomly, selecting in first place OD (Oculus Dexter) or OS (Oculus Sinister) in each subject. To improve fixation stability, sessions were broken into 47-s segments and 8 trials were recorded in total. The frame rate was 59.81 Hz, the amplifier gain 10^4^ and bandwidth 10–200 Hz. The signals have been digitized with a sampling rate of 1017 samples/sg, being 84 the number of samples in each recording (length 82.61 ms).

The mfERG recordings from OD eyes were used. Individual mfERG responses for the 61 hexagons were grouped all together (whole field: SUM), into five concentric rings centred on the fovea for analysis: R1 (0–1.75°, 16 degrees^2^, 1 hexagon), R2 (6.25°, 23 degrees^2^, 6 hexagons, perifoveal ring), R3 (11.5°, 36 degrees^2^, 12 hexagons), R4 (17.85°, 51 degrees^2^, 18 hexagons) and R5 (25.3°, 69 degrees^2^, 24 hexagons) (Fig 1A), in four quadrants (ST, IT, IN, SN) and in the nasal (NH), temporal (TH), superior (SH) and inferior (IH) hemifields (Fig 1B).

### Recordings’ analysis

In this study, two methods of analysis of the mfERG recordings are compared: a) standard method based on the amplitudes and latencies of N1 and P1 waves’ measuring, and b) method of averaging of the controls database.

### Standard method

**Fig 2:**
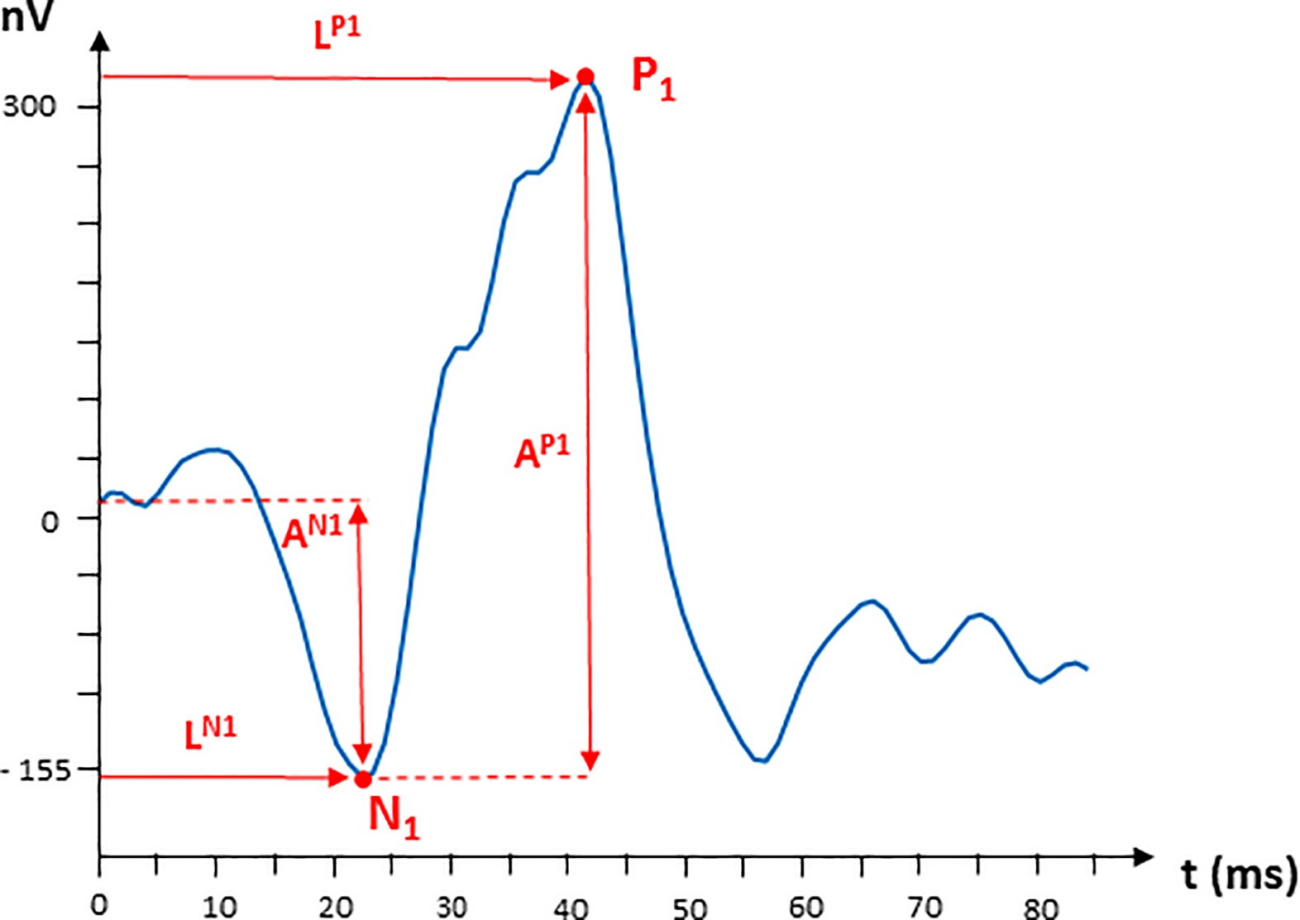
Waveform of first order kernel multifocal electroretinogram (mfERG) response (nV); SUM aggrupation of a control subject. Definition of the measurements of the parameters of waves N1 and P1.

Using functions we developed in Matlab, the amplitudes (A^N1^, A^P1^) and latencies of waves N1 and P1 (L^N1^, L^P1^) are calculated according to Hood et al. [5]. The location of N1 corresponds to the minimum recording inside the temporal window of 9 to 32 ms and P1 location is the maximum in the interval: (max{19, *L*^*N*1^} *to* 50) ms. The definition of the parameters is shown in Fig 2.

### Control recording averaging

The signals from OD eyes of 6 control subjects (*x*_1_,…*x*_*M*_ = 6) have been used to obtain a *model* signal of each mfERG averaging signal: sum, rings, quadrants and hemifields. The model signal y_M_ (k) is built averaging M = 6 signals in the temporal domain.

$$y_{M}(k)=\frac{1}{M}\sum_{i=1}^{M}x_{i}(k)\;(k=1\ldots .84)$$

**Fig 3:**
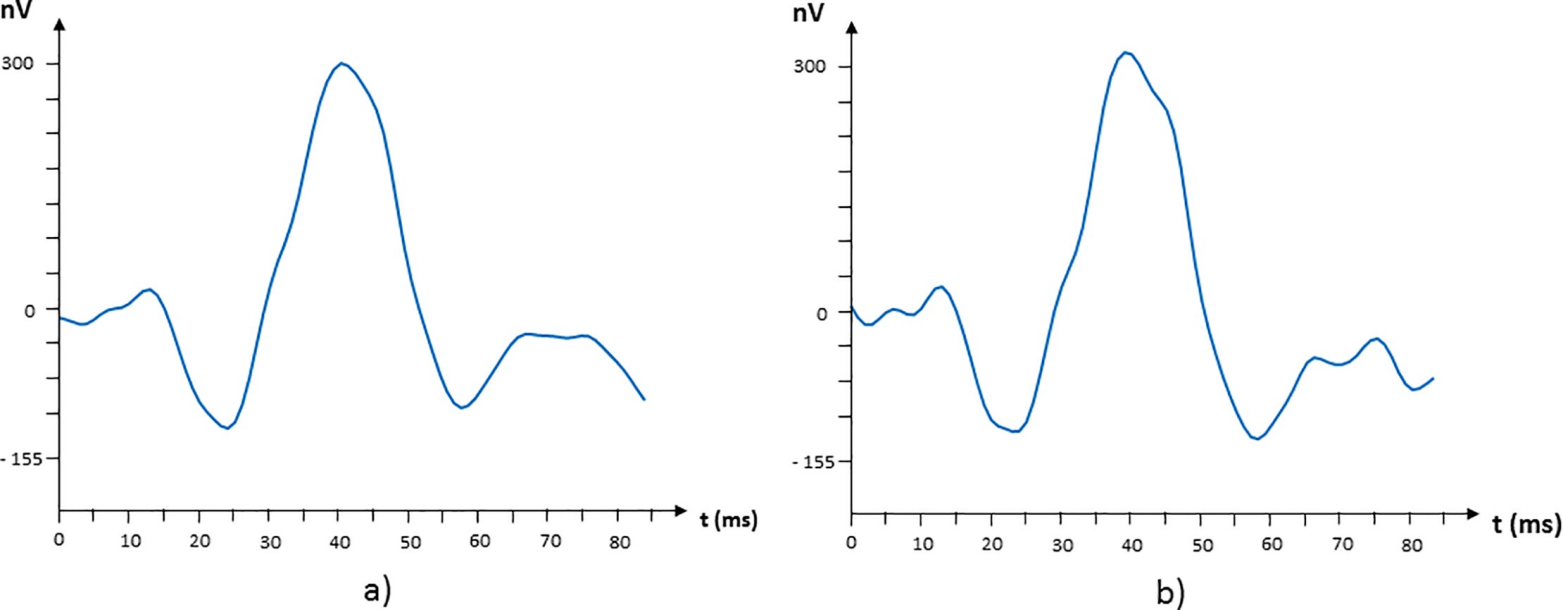
Model signals corresponding to the following regions: (a) R5 and (b) ST quadrant.

A model signal is obtained for each grouping under assessment: $y_{M}^{SUM}(k),\;y_{M}^{R1}(k),\ldots \;y_{M}^{R5}(k),y_{M}^{ST}(k),\ldots ,\;y_{M}^{SN}(k),\;y_{M}^{SH}(k),\ldots ,\;y_{M}^{NH}(k)$. In Fig 3 the averaging signals for the following groupings are shown: R5 and ST.

In order to characterize a signal in a defined group, it is compared to the model signal of that region using a standardized root-mean-square error (F_NRMSE_):

$$F_{NRMSE}=100\left(1-\frac{\left\|y_{M}-y\right\|}{\left\|y_{M}-mean(y_{M})\right\|}\right)$$

where *y*_*M*_ is the averaging signal and *y* the one whose evaluation is required. In case the fitting is perfect, F_NRMSE_ = 100.

### Statistical analysis

All statistical analyses were performed using the SPSS 25.0 software (SPSS Inc. Chicago, Illinois, USA) and STATGRAPHICS Centurion XVII. The results were expressed as the mean and the standard deviation. A p value below 0.05 was considered statistically significant.

The normality of the results was assessed using the Shapiro-Wilk (W) test. The differences between groups were evaluated using the independent t-test in normal distributions or the U-Mann-Whitney (Wilcoxon) test in non-normal distributions. The differences between methods were evaluated using the dependent t-test in normal distributions (paired-samples t-test).

## Results

### Standard method

**Fig 4:**
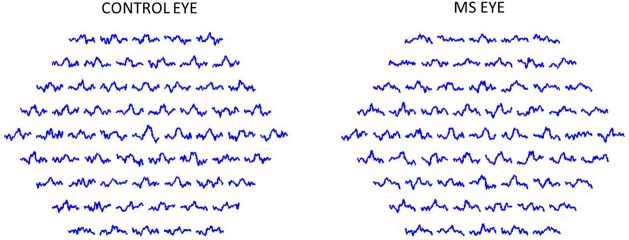
Multifocal electroretinogram (mfERG) first order kernel (nV) trace array obtained from a control subject (a) and a multiple sclerosis (MS) patient (b).

In Fig 4 mfERG signals (in amplitude units: nV) of a healthy control and a patient with MS are shown.

The mean values of amplitude and latency of N1 and P1 peaks obtained for each database are shown in Table 1 (standard method).

**Table 1:** Mean and standard deviation (SD) values of amplitudes and latencies in controls (C) and multiple sclerosis (MS) patients.

Region	A^N1^(nV)		L^N1^ (ms)		A^P1^ (nV)		L^P1^(ms)	
	C	MS	C	MS	C	MS	C	MS
**SUM**	160.41(53.87)	155.44(82.84)	24.75(0.74)	23.07(3.52)	469.09(134.02)	448.30(199.04)	42.77(2.13)	42.02(3.40)
**R1**	308.17(79.95)	533.49(456.84)	25.89(4.60)	26.22(4.18)	986.50(196.80)	1336.15(757.21)	49.00(0.40)	44.05(7.85)
**R2**	207.73(59.18)	274.29(130.37)	25.40(2.60)	22.16(4.94)	612.22(139.13)	780.36(264.38)	45.56(1.72)	42.74(3.86)
**R3**	179.97(45.95)	266.06(96.70)	24.25(1.48)	22.62(3.83)	517.28(101.32)	660.74(242.30)	41.79(2.55)	41.56(4.31)
**R4**	161.17(58.22)	201.03(92.54)	24.25(1.02)	22.16(3.42)	464.08(150.25)	530.63(199.18)	42.77(2.13)	43.13(2.65)
**R5**	158.06(63.45)	176.27(90.63)	23.60(2.06)	21.76(5.90)	458.38(131.61)	478.24(167.64)	42.45(2.28)	41.95(4.10)
**ST**	195.55(41.52)	228.98(200.02)	24.09(1.36)	22.22(3.73)	583.38(83.32)	613.89(341.15)	43.10(1.80)	42.41(3.40)
**IT**	148.12(67.56)	186.46(95.40)	24.42(1.80)	21.44(5.90)	425.81(161.75)	493.95(177.68)	41.63(3.15)	39.72(5.17)
**IN**	137.41(66.43)	258.50(136.53)	23.93(2.12)	23.21(4.28)	411.36(174.57)	596.14(249.49)	40.97(3.66)	42.02(3.25)
**SN**	188.45(70.19)	227.41(88.25)	23.76(1.15)	22.94(4.80)	524.81(137.57)	594.56(161.51)	42.94(1.72)	42.22(5.56)
**SH**	186.06(56.03)	186.20(73.51)	24.42(0.74)	22.03(3.95)	542.37(115.97)	531.38(181.87)	42.94(1.61)	40.31(6.74)
**IH**	139.34(62.06)	176.39(73.96)	24.09(1.73)	23.21(3.71)	409.97(160.61)	467.64(171.50)	41.46(3.20)	42.02(3.61)
**TH**	169.01(45.16)	175.22(71.61)	24.25(1.48)	21.76(4.89)	494.17(109.44)	503.73(180.31)	42.77(2.13)	40.90(4.53)
**NH**	159.17(64.36)	199.50(79.04)	23.76(1.15)	23.01(3.77)	454.31(152.16)	510.18(178.36)	42.94(1.93)	41.49(5.40)

A^N1^: amplitude of wave N1; L^N1^: latency of wave N1; A^P1^: amplitude of wave P1; L^P1^: latency of wave P1; C: controls; MS: patients; SUM: whole field; R1,…R5: Ring 1,…Ring 5; ST: superior temporal quadrant; IT: inferior temporal quadrant; IN: inferior nasal quadrant; SN: superior nasal quadrant; SH: superior hemifield; IH: inferior hemifield; TH: temporal hemifield; NH: nasal hemifield.

**Fig 5:**
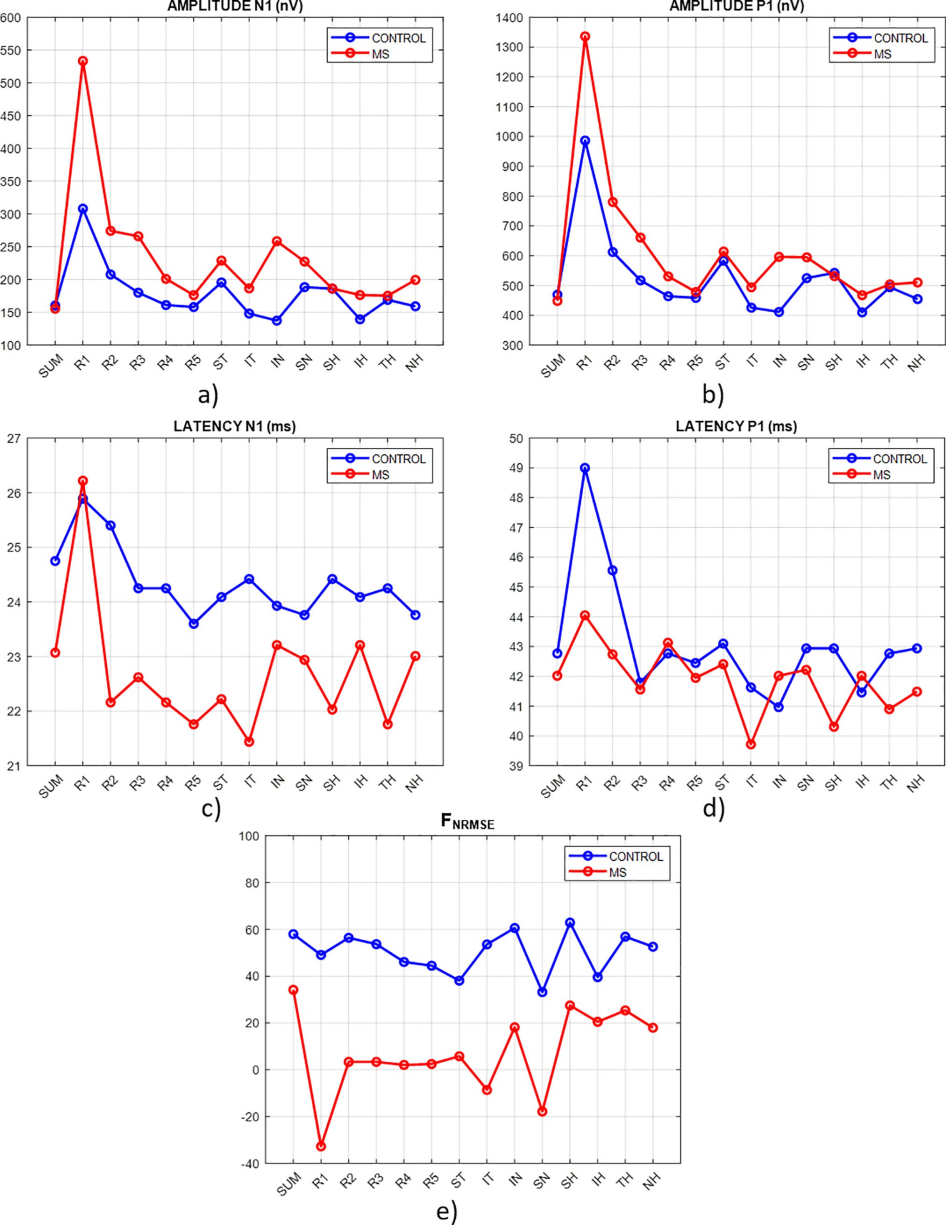
Principal results of the multifocal electroretinogram (mfERG) analysis.

The four parameters A^N1^, L^N1^, A^P1^ and L^P1^ present a Pearson correlation positive and significant (p<0.05) among themselves, being the highest correlation r = 0.867 between A^N1^ and A^P1^, and therefore being the behaviour in both amplitudes very similar, as it is shown in (Fig 5A and 5B). In the ring analysis, amplitudes A^N1^ and A^P1^ have a maximum value in R1 (fovea) and progressively decrease to the exterior (parafovea, perifovea) due to the existence of a maximum concentration of cones in the fovea that decreases towards the periphery [12]. Only amplitude N1 significantly discriminated between controls and MS patients (U Mann-Whitney test) in Ring3 (p = 0.023) and IN (p = 0.036).

There is no significant difference in any analysis regions between controls and MS in the N1 latency (U Mann-Whitney, p>0.095) and P1 amplitude (U Mann-Whitney, p>0.132) parameters.

The latency of wave P1 only presents significant differences between controls and MS in Ring1 (U Mann-Whitney, p = 0.018) (Fig 5C and 5D). L^P1^ presents a value significantly superior in the fovea, progressively decreasing to the parafovea and the perifovea in both MS and controls.

Table 2 shows F_NRMSE_ values in controls and MS patients. To obtain F_NRMSE_ values in the 6 control eyes, the averaging signal has been built by means of cross-validation in 6 folds (leave-one-out cross-validation: M = 5, the analysed signal is not used to construct the model). To obtain the F_NRMSE_ values of MS patients, the averaging signal has been implemented with all the eyes of controls (M = 6).

**Table 2:** Mean and standard deviation (SD) of normalized root-mean-square error function (F_NRMSE_) in controls and multiple sclerosis (MS) patients.

Region	Controls	MS	p value
**SUM**	58.0(16.9)	34.2(28.4)	0.086
**R1**	49.1(9.5)	-32.7(122.3)	0.014
**R2**	56.4(13.1)	3.4(70.6)	0.010
**R3**	53.7(12.5)	3.4(69.4)	0.018
**R4**	46.1(21.1)	2.1(59.5)	0.035
**R5**	44.5(14.9)	2.5(50.7)	0.036
**IN**	38.1(27.0)	5.8(56.5)	0.127
**SN**	53.6(16.9)	-8.6(91.9)	0.025
**ST**	60.6(12.2)	18.2(54.8)	0.013
**IT**	33.2(19.3)	-17.8(75.9)	0.097
**SH**	62.9(13.6)	27.5(35.3)	0.036
**IH**	39.6(22.8)	20.5(36.5)	0.087
**TH**	56.9(13.5)	25.4(31.5)	0.126
**NH**	52.6(21.5)	18.0(51.0)	0.045

SUM: whole field; R1,…R5: Ring 1,…Ring 5; ST: superior temporal quadrant; IT: inferior temporal quadrant; IN: inferior nasal quadrant; SN: superior nasal quadrant; SH: superior hemifield; IH: inferior hemifield; TH: temporal hemifield; NH: nasal hemifield.

F_NRMSE_ values in controls exceed significantly (t-student independent samples test, p<0.0001) (Fig 5E) the values obtained from MS signals, since the model signal is built from control signals. F_NRMSE_ presents a significant negative correlation with both amplitudes and latency of P1 wave. F_NRMSE_ regional behaviour can be seen in Fig 5E.

The capacity of discrimination between controls and MS for the standard analysis method and the averaging method is shown in Table 3.

**Table 3:** Measurements of area under ROC (receiver operating characteristic curve) (AUC) using standard method and normalized root-mean-square error function (F_NRMSE_) to discriminate between multiple sclerosis patients and controls.

Region	AUC values					
	Parameters				Methods	
	*A*^*N1*^	*L*^*N1*^	A^P1^	L^P1^	Mean by region (standard method)	F_NMRSE_
**SUM**	0.50	0.65	0.57	0.53	0.56	0.76
**R1**	0.54	0.51	0.64	0.83	0.63	0.69
**R2**	0.67	0.68	0.74	0.73	0.71	0.78
**R3**	0.82	0.64	0.71	0.53	0.68	0.80
**R4**	0.64	0.72	0.61	0.56	0.63	0.76
**R5**	0.54	0.54	0.56	0.53	0.54	0.81
**IN**	0.80	0.54	0.71	0.61	0.67	0.67
**SN**	0.66	0.55	0.59	0.56	0.59	0.80
**ST**	0.52	0.58	0.54	0.52	0.54	0.91
**IT**	0.61	0.61	0.60	0.60	0.61	0.74
**SH**	0.53	0.70	0.51	0.54	0.57	0.84
**IH**	0.64	0.53	0.58	0.59	0.58	0.67
**TH**	0.69	0.54	0.59	0.53	0.59	0.81
**NH**	0.56	0.62	0.51	0.59	0.57	0.71
**Mean by parameter**	0.62	0.60	0.60	0.59	0.60	0.76

A^N1^: amplitude of wave N1; L^N1^: latency of wave N1; A^P1^: amplitude of wave P1; L^P1^: latency of wave P1; SUM: whole field; R1,…R5: Ring 1,…Ring 5; ST: superior temporal quadrant; IT: inferior temporal quadrant; IN: inferior nasal quadrant; SN: superior nasal quadrant; SH: superior hemifield; IH: inferior hemifield; TH: temporal hemifield; NH: nasal hemifield.

In the study of amplitudes and latencies (standard method) and considering the average value in every possible region as criteria for comparability, the most discriminant parameter is the amplitude of N1 wave ($mean({AUC}^{A^{N1}})=0.62$). The visual field region analysed with greater AUC value in mean value is R2 (*mean*(AUC_R2_) = 0.71). As best option using the standard method, the maximum capacity of discrimination could be obtained just analysing the N1 amplitude in R3: (${AUC}_{R3}^{A^{N1}}=0.82$).

F_NRMSE_ value synthesizes in just one parameter the comparison of the shapes of the wave, against the 4 parameters used in the traditional method. In the study of the different regions, in all cases the AUC value with F_NRMSE_ parameter exceeds or evens (IN region) the mean value in the standard method.

The mean value of AUC values using F_NRMSE_ parameter is 0.76 ($mean({AUC}^{F_{NRMSE}})=0.76$) in comparison with the average value obtained with the standard method: mean(AUC^STANDARD^) = 0.60). The greatest discriminant value is obtained in the ST quadrant analysis $\left({AUC}_{ST}^{F_{NRMSE}}=0.91\right)$ and the SH hemisphere $\left({AUC}_{SH}^{F_{NRMSE}}=0.84\right)$.

## Discussion

In this study, the differences in mfERG signals in control subjects and MS patients have been analysed using the standard method of measure for amplitudes and latencies of N1 and P1 waves, as well as a method based on the comparison of recordings with a reference template obtained from control signals.

There are few previous studies which analyse mfERG signals in patients with MS, and due to its heterogeneity (different types of patients, acquisition systems and analysis of recordings, reduced databases, etc.) the results are contradictory. On the other hand, in our study the most innovative and ambitious aspect is the use of patients with an early diagnosis (less than 6 months from their first symptom) and with no symptoms of visual acuity loss nor optic neuritis, since our aim was to assess the effects of the disease themselves and the existence of axonal degeneration and the affectation of the nerve impulse conduction in the visual canal since the beginning of the pathology. For this reason, we avoided patients with previous optic neuritis, in order to avoid biases including eyes in which inflammation of the optic nerve or visual atrophy can affect the nervous conduction of the visual channel significantly.

Gundogan et al [13] reported that there was no significant difference in P1 and N1 amplitude and implicit times in different rings, between patients with MS (without a history of optic neuritis) and control subjects. Nevertheless, in Saidha et al [14] it is valued that all waves of the first-kernel order showed normal latencies in all MS patients but a reduction of P1 amplitude is detected in 5 of 7 patients with predominant macular thinning. In Neroev et al [15] the use of the wave P1 latency in the parafovea as a marker of MS progression is suggested. Recently Hanson et al [16] observed that in a typical MS cohort, latency of wave P1 increases (in ring 2) whereas there is no difference observed in amplitudes.

In the present study, it can be observed that on average the most discriminant parameter to detect signals from MS patients is the amplitude of the N1 wave ($mean({AUC}^{A^{N1}})=0.62$). The most valuable region of the visual field for discriminating between controls and MS patients is ring R2 (*mean*(AUC_R2_) = 0.71). As a general idea, in the standard method the capacity of discrimination is low (AUCs always below 0.71).

In the second part of our study, the use of an averaging method is proposed to generate a signal template of controls which can be used to verify the degree of fitting, evaluated by means of mean squared error. F_NRMSE_ in controls is superior to MS patients (p<0.0001). With this new method, we observe that AUC between healthy controls and MS patients increases in most regions of the retina analysis (except in region IN). An advantage of the F_NRMSE_ parameter is that this one models the mfERG signal form through a characteristic that defines the global wave form of the acquired signals, in comparison with the control subject signals.

With the obtained results of the averaging parameter F_NRMSE,_ we have observed that the greatest discriminating power is produced in the ST region (${AUC}_{ST}^{F_{NRMSE}}=0.91$), improving results obtained in the analysis of amplitudes and latencies. Previous studies carried out with OCT also point to temporal sectors as the ones which present an early affectation at early stages of the disease [17].

Our population was composed of patients with early-stage MS, so markedly delayed transmission of nerve stimulation was not yet apparent. We also detected more subclinical alterations in mfERG amplitudes than in mfERG latencies. Probably, when MS progresses in our population or MS outbreaks causes myelin loss, we will detect more alterations in mfERG latencies.

In our patients, we found affectation in the superior temporal quadrant. This concurs with the findings of previous authors who demonstrated early damage in the temporal RNFL quadrant using OCT [18]. This temporal quadrant corresponds topographically to the papillomacular bundle and is formed by the superior temporal and inferior temporal sectors. Early neurodegeneration of the RNFL usually begins in the thickest superior temporal sector. This occurs not only in MS [19] but also in other neurodegenerative processes such as glaucoma [20,21], Parkinson’s disease [22] and Alzheimer’s disease [23].

In conclusion, the correct analysis of mfERG recordings can be an effective biomarker for diagnosis of MS in subjects at early diagnosis and without previous history of optic neuritis, which helps to accelerate the diagnosis and to apply the treatment from the early stages of the pathology, avoiding the reduction of patients’ quality of life and the appearance of new flare-ups.

The main limitation of our study is the small size of our database. In spite of this, we found significant differences between control subjects and patients, suggesting that the diagnostic capacity of the method may increase with sample size. Another limitation is that follow-up of these patients and controls was not carried out. To avoid bias derived from differences between databases, these should be extended to include subjects from other centres and signals recorded using other commercially available equipment. In addition, this study must be extended to other types of MS because so far just the Relapsing Remitting type has been studied.

## Supporting information

S1 Dataset Minimal Dataset (Minimal data set). (XLSX)
